# Clinical Significance of JAM‐2 Expression in the Vaginal Wall Tissues of Patients With Pelvic Organ Prolapse

**DOI:** 10.1111/jcmm.70512

**Published:** 2025-03-30

**Authors:** Yuan Liu, Yajing Mao, Yuelin Wu, Sheng Wan, Shengyi Gu, Jing Peng, Bo Jiao, Xiaolin Hua

**Affiliations:** ^1^ The International Peace Maternity & Child Health Hospital of China Welfare Institute (IPMCH) Shanghai Jiao Tong University School of Medicine Shanghai China; ^2^ Department of Obstetrics, Shanghai First Maternity and Infant Hospital, School of Medicine Tongji University Shanghai China; ^3^ Shanghai Key Laboratory of Maternal Fetal Medicine, Shanghai First Maternity and Infant Hospital, School of Medicine Tongji University Shanghai China; ^4^ Clinical Research Unit, Shanghai First Maternity and Infant Hospital, School of Medicine Tongji University Shanghai China; ^5^ Jiading Maternal and Child Health Hospital Shanghai China; ^6^ Hainan Branch, Shanghai Children's Medical Center, School of Medicine Shanghai Jiao Tong University Sanya China

**Keywords:** collagen I, junctional adhesion molecule 2 (JAM‐2), matrix metalloproteinase 2 (MMP‐2), pelvic organ prolapse (POP)

## Abstract

This study aimed to elucidate the roles of junctional adhesion molecule 2 (JAM‐2), collagen I and matrix metalloproteinase 2 (MMP‐2) in the pathogenesis of pelvic organ prolapse (POP) and explore their potential as diagnostic markers. We examined 82 POP patients and 64 controls using enzyme‐linked immunosorbent assay (ELISA) and quantitative Polymerase Chain Reaction (qPCR) to analyse protein and gene expression levels of JAM‐2, Collagen I and MMP‐2. Receiver operating characteristic (ROC) analysis evaluated their diagnostic efficacy, with correlation analyses linking molecular markers to POP severity based on POP‐Q grades. Our study found no significant differences in age, BMI and vaginal parity between POP patients and controls. Molecular analyses revealed significant alterations in the expression levels of JAM‐2, Collagen I and MMP‐2 in POP patients. Specifically, there was a marked decrease in JAM‐2 and collagen I levels, accompanied by an increase in MMP‐2 expression, indicating a disruption in the balance between tissue synthesis and degradation. ROC analysis demonstrated the significant discriminative power of these markers, with substantial area under the curve (AUC) values for diagnosing POP. Correlation analysis further showed a significant association between the expression of JAM‐2, Collagen I and MMP‐2 and the clinical severity of POP, as indicated by POP‐Q grades. Our findings revealed the significant changes in the expression of JAM‐2, Collagen I and MMP‐2 that may contribute to the POP pathogenesis. The diagnostic potential of these markers was substantiated, suggesting their utility in developing noninvasive diagnostic tools for POP.

## 1 Introduction

Pelvic organ prolapse (POP) represents a significant clinical challenge within the realm of pelvic floor dysfunction (PFD), a complex condition arising from the weakening of support tissues in the pelvic floor [1]. This weakening leads to the displacement and malfunctioning of pelvic organs, manifesting primarily as uterine prolapse and relaxation of the anterior and posterior vaginal walls, among other forms of prolapse, as well as stress urinary incontinence (SUI) [1, 2, 3]. The occurrence of PFD is closely tied to the mechanical properties of vaginal and pelvic floor‐supportive tissues [2, 4, 5, 6]. PFD is characterised by extracellular matrix alterations, particularly in collagen composition, which contribute to tissue damage and structural compromise. With an ageing global population, the impact of POP on the quality of women's lives, including its psychological burdens, has garnered increasing attention from healthcare researchers worldwide. Despite the critical need, traditional surgical interventions have often fallen short of expectations. While pelvic floor reconstructive surgeries have improved anatomical outcomes, postoperative complications remain a significant concern. Consequently, there is an urgent demand for a safe, effective, minimally invasive approach that addresses the pathogenesis of PFD directly.

Structural changes in extracellular matrix (ECM) components, particularly collagen and elastic fibres produced by fibroblasts, significantly reduce the tensile strength of pelvic floor support tissues, contributing to PFD [7]. The ECM plays a crucial role in chemical synapse signalling junctions, serving not only as a structural framework that preserves the spatial relationship between motor neurons and muscle cells. In connective tissue, the ECM is plentiful, providing substantial mechanical support and buffering cells from experiencing high pressure [8]. Recent research has illuminated the mechanisms by which ECM alterations contribute to PFD [4, 7]. Studies have shown that in individuals with PFD, there is a marked degradation of collagen and elastin fibres, which compromises the structural integrity and mechanical properties of the pelvic floor [9, 10]. This degradation is often mediated by an imbalance between the synthesis and degradation of ECM components [4], influenced by factors such as ageing, hormonal changes, childbirth and genetic predisposition. Specifically, an increase in the activity of matrix metalloproteinases (MMPs), enzymes responsible for the degradation of ECM proteins, has been observed in PFD patients. This enzymatic activity, particularly that of MMP‐2, has been linked to the disruption of collagen and elastin fibres [11, 12], contributing to the weakening of the pelvic floor support system.

The JAM protein family comprises four primary members: JAM‐A, JAM‐B, JAM‐C and JAM‐L, and is found at cell–cell junctions and is particularly concentrated in tight junctions (TJs) through homotypic interactions [13]. Predominantly, JAM‐B, also named JAM‐2, is expressed in the heart, endothelial cells, placental trophoblasts, high endothelial venules and arteriole endothelium. Its interaction with the ECM is mediated through its association with integrins and other cell surface proteins that directly bind to ECM components [14]. Functionally, JAM‐2 is involved in several key processes, including the regulation of paracellular permeability in endothelial and epithelial cells, recruitment of leucocytes in inflammation, angiogenesis and the promotion of cell growth and movement [13].

Our research aims to bridge a critical knowledge gap by exploring the association between JAM‐2 expression and the structural and mechanical integrity of pelvic floor support tissues. We hypothesised that lower expression levels of JAM‐2 in the vaginal wall tissues of POP patients may be linked to the observed degradation of collagen and the activity of MMPs, particularly MMP‐2, which are known to play a pivotal role in ECM remodelling and the pathogenesis of PFD [15, 16]. By examining the expression patterns of JAM‐2 and its correlation with collagen and MMP‐2 expression in the context of POP, our study seeks to elucidate the underlying molecular mechanisms that contribute to the weakening of pelvic floor support tissues.

## 2 Methods

### 2.1 Study Population and Design

This study was conducted on patients who presented to our department and underwent surgery for POP or for other gynaecological conditions (controls). The POP group included patients who underwent anterior vaginal wall repair or anterior pelvic floor reconstruction surgery due to anterior vaginal wall prolapse, with the severity assessed using the Pelvic Organ Prolapse Quantification system (POP‐Q), focusing primarily on uterine prolapse grades and accompanying degrees of anterior and posterior vaginal wall prolapse. Controls were selected from the same age group undergoing hysterectomy for conditions other than POP, excluding those with premalignant or early‐stage cervical cancer and PFD.

Informed consent was obtained from all participants prior to the surgery, and the study was approved by Shanghai First Maternity and Infant Hospital. The study procedures for the POP group involved either anterior vaginal wall repair or anterior pelvic floor reconstruction surgery. Control group surgeries were performed via vaginal or abdominal hysterectomy. During the surgeries, a full‐thickness tissue sample approximately 1.0 cm × 1.0 cm was excised from the anterior vaginal wall near the vault, immediately divided into two portions: one portion was rinsed with saline, placed in a sterile, enzyme‐free EP tube and quickly frozen in liquid nitrogen before being transferred to a −80°C freezer for storage; the other portion was fixed in 10% formalin solution for at least 24 h, then processed through dehydration, paraffin embedding and stored for further analysis.

### 2.2 Inclusion Criteria

All patients were postmenopausal and had not received systemic hormonal treatments within 3 months prior to surgery. Patients with neurological, respiratory diseases, functional ovarian tumours, metabolic diseases, connective tissue disorders, previous pelvic floor surgeries, urinary or genital tract infections or pelvic deformities were excluded from the study.

### 2.3 The Enzyme‐Linked Immunosorbent Assay (ELISA)

Tissue samples collected during surgery were lysed to prepare for protein analysis. The protein concentration of JAM‐2, Collagen I and MMP‐2 was determined using specific ELISA kits according to the manufacturer's instructions. The Human JAM‐B ELISA Kit (ab277453, Abcam, USA), Human COL1A1 (Collagen I) ELISA Kit (RK01149, ABclonal, USA) and Human MMP‐2 ELISA Kit (ab100606, Abcam, USA) were utilised. Sample absorbances were measured using a microplate reader, and protein concentrations were calculated based on standard curves established with known concentrations of each protein.

### 2.4 Real‐Time Quantitative Reverse Transcription PCR (qRT‐PCR)

Total RNA was extracted from the tissue samples using a standardised protocol to assess the mRNA expression levels of JAM‐2, COL1A1 and MMP‐2. Reverse transcription was performed to synthesise cDNA from the total RNA using a kit (TIANGEN, China). Quantitative Real‐Time PCR (qRT‐PCR) was then carried out using the Quant One‐Step qRT‐PCR Kit (TIANGEN) with specific primers for JAM‐2 (Forward GTGGCCTTGGTGTATGCTAT, Reverse TCACTCATTGTCGTGGCTTT), COL1A1 (Forward GTTGTGCGATGACGTGATCTGTGA, Reverse TTCTTGGTCGGTGGGTGACTCTG), MMP‐2 (Forward TACAGGATCATTGGCTACACACC, Reverse GGTCACATCGCTCCAGACT) and GAPDH (Forward ACAGTCAGCCGCATCTTCT, Reverse ACTCCGACCTTCACCTTCC) as the internal control. The relative expression levels of the target genes were calculated using the 2^(‐ΔΔCt)^ method, normalising against GAPDH expression.

### 2.5 Statistical Analysis

Statistical analysis was conducted using SPSS version 25.0. Continuous variables, such as age, BMI and expression levels of JAM‐2, Collagen I and MMP‐2, were presented as mean ± standard deviation (SD). Comparisons between POP and control groups were made using Student's t‐test or Mann–Whitney U test for nonnormally distributed data, while categorical variables like vaginal parity were analysed using chi‐square or Fisher's exact tests. The diagnostic efficacy of biomarkers was assessed through receiver operating characteristic (ROC) curve analysis, providing area under the curve (AUC), sensitivity, specificity and Youden index. Spearman's rank correlation coefficient analysed the correlation between biomarker levels and POP‐Q grades, with Pearson correlation for biomarker interrelationships. A *p*‐value < 0.05 was considered statistically significant.

## 3 Results

### 3.1 Demographic and Clinical Profile of POP Patients and Controls

This study included 82 patients with POP who underwent either anterior vaginal wall repair or anterior pelvic floor reconstruction surgery due to anterior vaginal wall prolapse (Table 1). The mean age and body mass index (BMI) were not significantly different between the groups, with *p*‐values of 0.272 and 0.179, respectively. Regarding vaginal parity, the distribution within the control group was as follows: 7 (10.9%) had a vaginal parity of 0, 32 (50%) had a parity of 1, 17 (26.6%) had a parity of 2, and 8 (12.5%) had a parity of more than 2. In the POP group, 3 (3.6%) had a vaginal parity of 0, 35 (42.7%) had a parity of 1, 29 (35.4%) had a parity of 2, and 15 (18.3%) had a parity of more than 2. The lack of statistically significant differences in age, BMI and vaginal parity suggests that these factors are not strongly associated with the presence of POP in this particular study population.

**TABLE 1:** Demographic and clinical characteristics of patients with pelvic organ prolapse (POP) and controls.

Characteristics	Study groups		*p*
	Controls (*n* = 64)	POP (*n* = 82)	
Age (years)	57.95 ± 9.82	59.36 ± 11.04	0.272
Body mass index (kg/m^2^)	23.39 ± 4.28	24.73 ± 4.41	0.179
Vaginal party			
0	7 (10.9%)	3 (3.6%)	0.183
1	32 (50%)	35 (42.7%)	
2	17 (26.6%)	29 (35.4%)	
> 2	8 (12.5%)	15 (18.3%)	

*Note:* Values were presented as mean ± SD or *n* (percentage, %). *p*‐values were derived from Mann–Whitney test. Chi‐square test was used for assessing distribution of observations or phenomena between the two groups.

### 3.2 Expression Patterns of JAM‐2, Collagen I and MMP‐2

**FIGURE 1:**
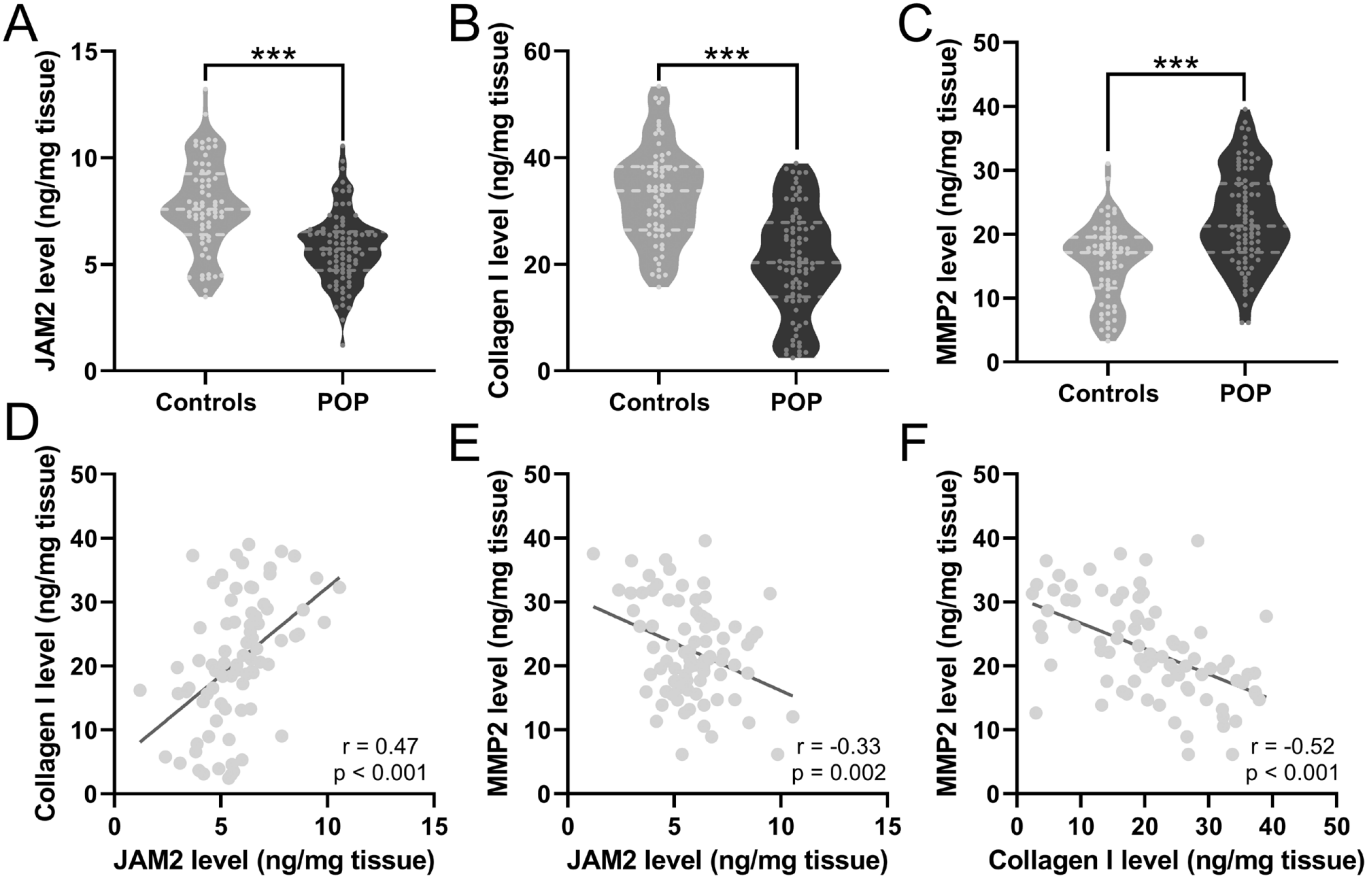
Comparisons of JAM2 (A), Collagen I (B) and MMP2 (C) levels in vaginal wall tissues between patients with pelvic organ prolapse (POP, *n* = 82) and controls (*n* = 64). Violin plot, ***p < 0.001 from Unpaired t‐test with Welch's correction. Pearson correlation analysis of JAM2 and Collagen I levels (D), JAM2 and MMP2 levels (E), Collagen I and MMP2 levels (F) in vaginal wall tissues from patients with pelvic organ prolapse (POP, *n* = 82).

To elucidate the molecular underpinnings of POP, we embarked on a comparative study to evaluate the expression levels of JAM‐2, Collagen I and MMP‐2, which are integral to the structural integrity of vaginal wall tissues. Our analyses revealed a marked reduction in the protein levels of JAM‐2 and Collagen I in the POP group, in stark contrast to the significantly elevated levels of MMP‐2 (Figure 1A–C). This disparity suggests a perturbation in the connective tissue maintenance and reparative mechanisms within the pelvic floor matrix. A robust Pearson correlation analysis reinforced these findings, showing a significant positive correlation between JAM‐2 and Collagen I levels, signalling a coupled decline of these structural proteins as JAM‐2 decreases (*p* < 0.001, Figure 1D). Conversely, a significant negative correlation emerged between JAM‐2 and MMP‐2 (*p* = 0.002, Figure 1E), and Collagen I and MMP‐2 levels (*p* < 0.001, Figure 1F), hinting at a complex interplay where an increase in matrix degradation is concomitant with the downregulation of key structural components.

**FIGURE 2:**
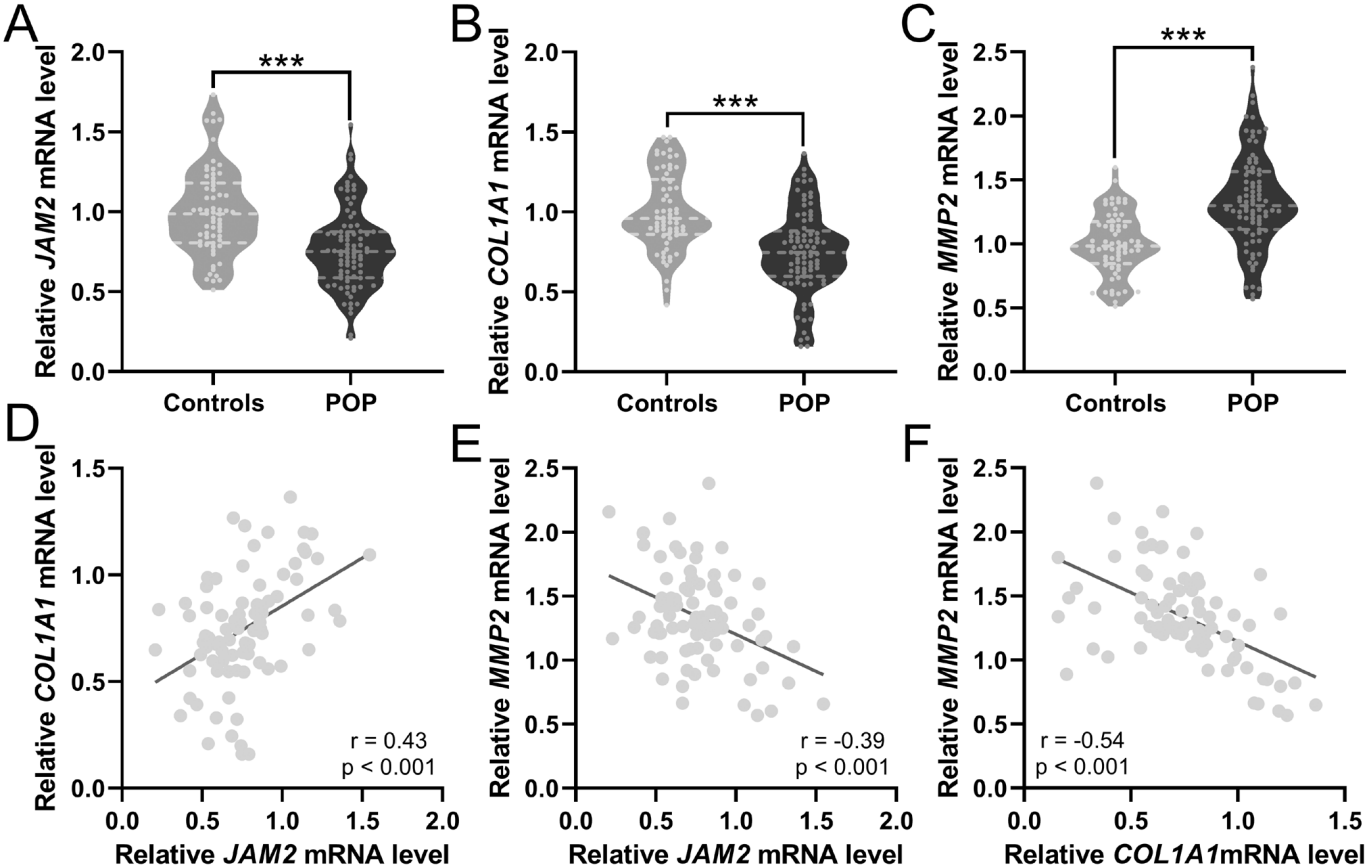
Comparisons of mRNA expressions of JAM2 (A), Collagen I (B) and MMP2 (C) levels in vaginal wall tissues between patients with pelvic organ prolapse (POP, *n* = 82) and controls (*n* = 64). Violin plot, ***p < 0.001 from Unpaired t‐test with Welch's correction. Pearson correlation analysis of JAM2 and Collagen I mRNA expressions (D), JAM2 and MMP2 mRNA expressions (E), Collagen I and MMP2 mRNA expressions (F) in vaginal wall tissues from patients with pelvic organ prolapse (POP, *n* = 82).

In parallel, the gene expression assessment of these molecules echoed the protein expression trends. There were significant downtrends in the mRNA expressions of JAM‐2 and Collagen I, accompanied by a notable uptick in MMP‐2 expression within the POP contingent (Figure 2A–C). Correlation analysis not only corroborated these patterns but also quantified the relationships, showcasing a significant positive relationship between JAM‐2 and Collagen I mRNA expressions (*p* < 0.001, Figure 2D). The relationship between JAM‐2 and MMP‐2 (*p* < 0.001, Figure 2E), as well as between Collagen I and MMP‐2 (*p* < 0.001, Figure 2F), was significantly negative, reinforcing the protein data. These insights into gene and protein expression profiles furnish a clearer understanding of the molecular alterations present in POP and could potentially inform targeted therapeutic strategies.

### 3.3 Diagnostic Efficacy of JAM‐2, Collagen I and MMP‐2 for Pelvic Organ Prolapse

**FIGURE 3:**
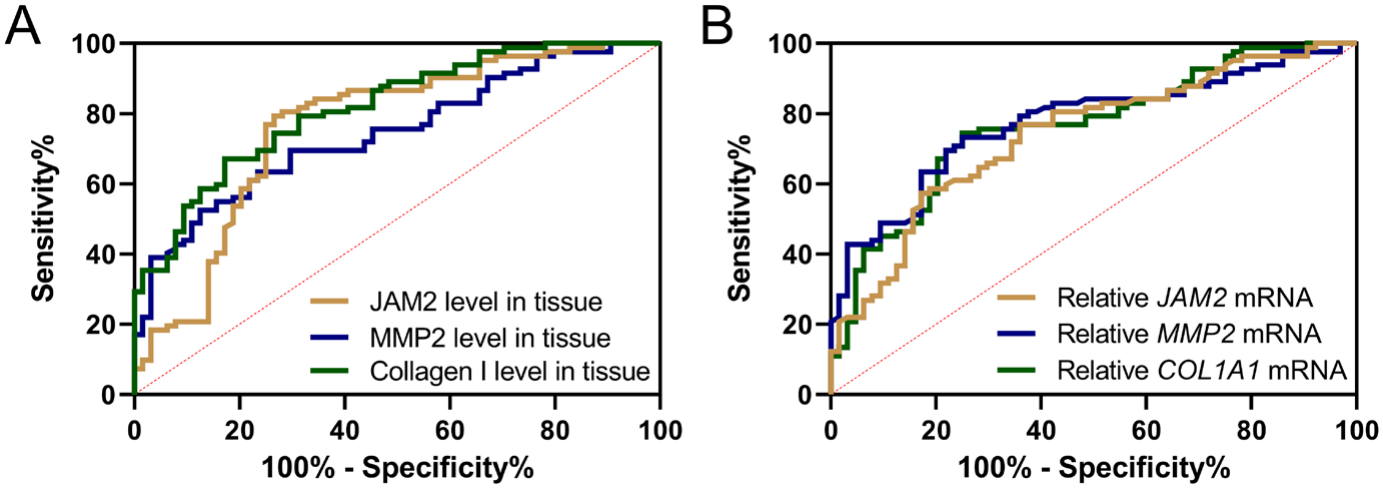
ROC analysis of JAM2, Collagen I and MMP2 levels in vaginal wall tissues (A) and mRNA expressions of JAM2, Collagen I and MMP2 levels in vaginal wall tissues (B) for the distinguishing of pelvic organ prolapse from controls.

We next performed the Receiver Operating Characteristic (ROC) analysis to evaluate the diagnostic value of JAM‐2, Collagen I and MMP‐2 for differentiating between patients with POP and controls. It was observed that the Area Under the Curve (AUC) values for the protein levels of JAM‐2, Collagen I and MMP‐2 in tissue were 0.77, 0.82 and 0.75 respectively (*p* < 0.001), suggesting significant discriminative power (Figure 3A and Table 2). The sensitivity and specificity percentages for JAM‐2 were 79.27% and 73.44%, with a Youden index of 0.53, indicating a moderate level of diagnostic accuracy. Collagen I showed a higher level of specificity at 82.81% and a sensitivity of 67.07%, yielding a Youden index of 0.49. MMP‐2 had a sensitivity of 69.51% and a specificity of 70.31%, with a Youden index of 0.39, demonstrating its utility in distinguishing POP from non‐POP conditions, albeit with slightly less accuracy than JAM‐2 and Collagen I. The mRNA expressions of JAM‐2, Collagen I (COL1A1) and MMP‐2 yielded AUC values of 0.74, 0.76 and 0.77, respectively (*p* < 0.001, Figure 3B and Table 2), indicating a substantial predictive value. The sensitivity and specificity of JAM‐2 mRNA expression were 76.83% and 64.06%, respectively, with a Youden index of 0.41. The mRNA expression of Collagen I showed a sensitivity of 74.39% and a specificity of 75.00%, resulting in a Youden index of 0.49. MMP‐2 mRNA expression demonstrated similar diagnostic efficacy with a sensitivity of 73.17% and a specificity of 75.00%, and a Youden index of 0.48.

**TABLE 2:** Predictive values in the ROC analysis.

	AUC	95% CI	*p*	Sensitivity (%)	Specificity (%)	Youden index
JAM2 level in tissue	0.77	0.68–0.85	< 0.001	79.27	73.44	0.53
Collagen I level in tissue	0.82	0.75–0.88	< 0.001	67.07	82.81	0.49
MMP2 level in tissue	0.75	0.67–0.82	< 0.001	69.51	70.31	0.39
Relative JAM2 mRNA	0.74	0.66–0.82	< 0.001	76.83	64.06	0.41
Relative COL1A1 mRNA	0.76	0.69–0.84	< 0.001	74.39	75.00	0.49
Relative MMP2 mRNA	0.77	0.69–0.85	< 0.001	73.17	75.00	0.48

Abbreviation: CI, confidence interval.

### 3.4 Correlation of POP‐Q Grade With JAM‐2, Collagen I and MMP‐2 Expression in POP

**FIGURE 4:**
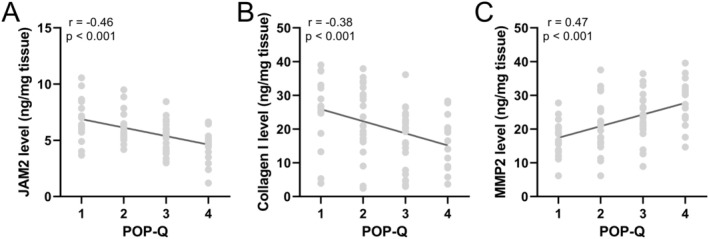
Spearman correlation analysis of POP‐Q grade with JAM2 (A), Collagen I (B) and MMP2 (C) levels in vaginal wall tissues from patients with pelvic organ prolapse (POP, *n* = 82).

**FIGURE 5:**
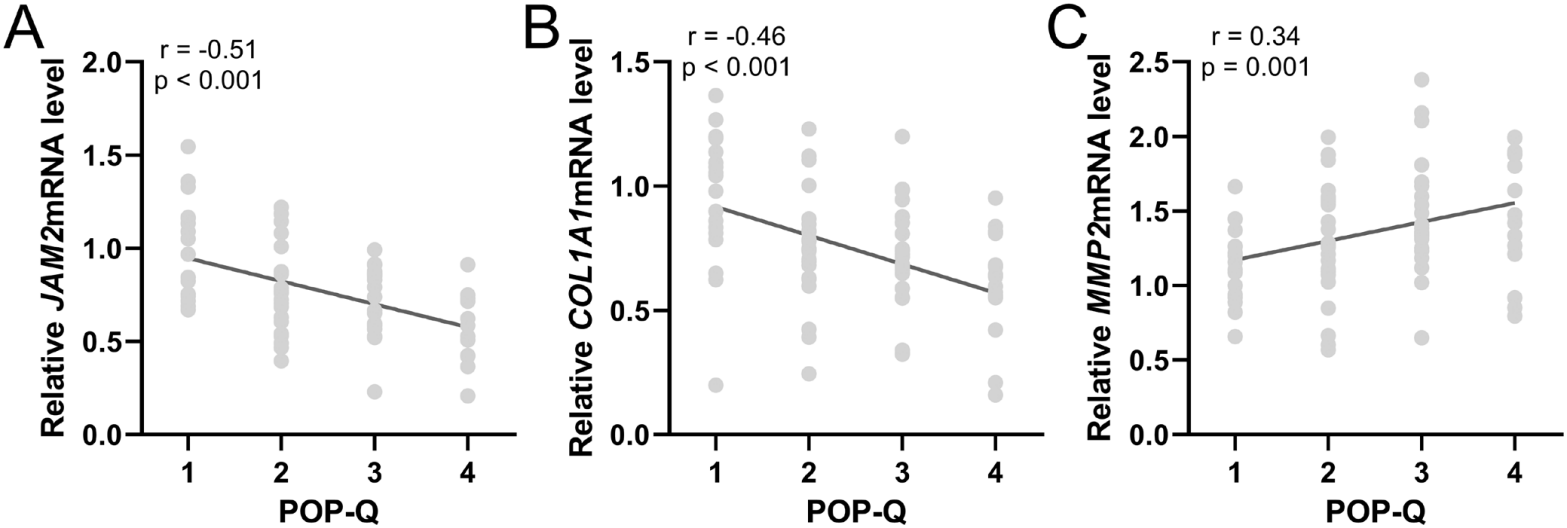
Spearman correlation analysis of POP‐Q grade with mRNA expressions of JAM2 (A), Collagen I (B) and MMP2 (C) levels in vaginal wall tissues from patients with pelvic organ prolapse (POP, *n* = 82).

To further evaluate the correlation between the expression of JAM‐2, Collagen I and MMP‐2 and the grade of pelvic organ prolapse (POP‐Q), we employed Spearman correlation analysis and found that as the severity of the disease increased, the concentration of JAM‐2 and Collagen I in the vaginal wall tissues notably decreased (Figure 4A,B), while the concentration of MMP‐2 correspondingly increased (Figure 4C), suggesting a significant correlation between the progression of POP and the alteration of these protein levels. Similarly, an analysis of mRNA expressions in relation to POP‐Q grades was performed, revealing that the expressions of JAM‐2 and Collagen I genes were significantly reduced (Figure 5A,B), whereas the expression of the MMP‐2 gene was increasingly pronounced with the advancing severity of POP (Figure 5C). This trend was consistent across the 82 patients, reflecting a direct association between the molecular expression patterns and the clinical grading of the disease.

## 4 Discussion

Our study provides novel insights into POP, expanding the current understanding of the condition. We observed significant changes in the expression of JAM‐2, Collagen I and MMP‐2, revealing a disrupted balance between tissue repair and degradation. Interestingly, this molecular landscape proved more informative than traditional risk factors such as age, BMI and vaginal parity. Furthermore, our ROC analysis demonstrated the substantial diagnostic potential of these molecular markers, suggesting a revolutionary approach to POP diagnosis and laying the groundwork for noninvasive diagnostic techniques. This innovative perspective not only deepens our comprehension of POP but also opens avenues for novel treatment strategies focused on restoring the integrity and functionality of the pelvic floor.

The ECM is composed of two main categories of biomolecules: fibrous proteins, such as collagen, elastin, fibronectin and laminins. These biomolecules provide structural integrity and mechanical properties, and proteoglycans, which regulate tissue biomechanics. Previous studies have identified various genes linked to POP, highlighting the role of ECM pathways in this condition. These genes encompass COL1A1, COL3A1, laminin gamma‐1 (LAMC1) and MMPs, all of which are integral to ECM structure and function [17, 18, 19, 20, 21]. Further analyses have identified genetic polymorphisms affecting POP, particularly in genes related to steroid hormone receptors and collagen/elastic fibre synthesis [20]. This suggests that defects in ECM synthesis and metabolism could undermine the pelvic support system. Therefore, degradation of ECM frequently weakens supportive structures, playing a significant role in the development of POP. Despite these findings, the precise molecular processes responsible for ECM degradation remain to be fully elucidated.

Our investigation provided an unprecedented look into the molecular dynamics of POP, focusing on the pivotal roles of JAM‐2, Collagen I and MMP‐2. JAM‐2 is fundamental for cell–cell adhesion, playing a significant role in maintaining tissue architecture and integrity. Although JAMs are not ECM proteins but are integral membrane proteins that belong to the immunoglobulin superfamily [22], they are primarily associated with TJs in epithelial and endothelial cells. Additionally, JAMs play key roles in the regulation of paracellular permeability, maintenance of cell polarity and various signalling pathways that influence cell behaviour. JAMs contribute to the organisation and function of the cell–cell junctions that are critical for the integrity and barrier function of tissues. JAMs interact closely with ECM components and cellular receptors to mediate cell adhesion, migration and inflammation, influencing the structural organisation of ECM and signalling functions [23]. For example, JAMs interact with integrins, which are critical receptors for ECM proteins, facilitating the communication between cells and the ECM [23]. Therefore, while JAMs are not ECM proteins, they play a significant role in the dynamic interplay between cells and the ECM. We observed pronounced reductions in JAM‐2 expression among POP patients. This is the first study showing a correlation of JAM‐2 with POP.

Collagen I, the most abundant collagen in the human body, is essential for providing strength and structural support to connective tissues, including the vaginal wall [24]. Research on collagen I levels in POP patients has yielded conflicting results. Some studies have documented a notable reduction in collagen I in the vaginal wall, uterosacral and cardinal ligaments of POP patients; others have found no notable differences compared to controls [9, 25]. This inconsistency is also observed in tissue analyses from the same patient, with no differences in collagen levels between prolapsed and nonprolapsed tissues. Additionally, investigations using primary cultured fibroblasts from pelvic tissues have reported both an increase in collagen fibres in POP patients and significantly lower procollagen expression. These divergent findings suggest that the relationship between collagen I and POP may be influenced by factors beyond simple quantitative measures of collagen presence. Our findings add crucial evidence to this puzzle, emphasising the significance of collagen I alteration in the structural integrity of pelvic tissues and their susceptibility to prolapse. This highlights the urgent need for more nuanced investigations into how collagen I structural and functional properties are altered in POP, which could unveil new targets for therapeutic intervention and improve our understanding of the underlying mechanisms of POP.

MMPs including MMP‐2 are pivotal in ECM protein degradation, facilitating tissue remodelling. MMPs were initially found to be primarily associated with ECM composition regulation and cell migration by dismantling physical barriers like collagen [26]. Dysregulation of MMPs could result in tissue weakening and prolapse. We found that both translational and transcriptional levels of MMP‐2 were elevated in POP patients. Our results resonate with previous findings. For example, a prior study observed an increase in MMP‐2 level in the uterosacral ligament of primigravida women undergoing caesarean sections for obstructive labour during stages 1 and 2 [11]. Additionally, a direct link between heightened MMP‐2 expression and uterine prolapse was found, suggesting that variations in MMP‐2 levels contribute to the onset and progression of POP [27]. The correlation of POP‐Q grades with JAM‐2, Collagen I and MMP‐2 expressions provided critical insights into the progression of POP. Our study underscores the critical role of MMP‐2 alterations in the pathophysiology of POP, revealing significant changes in protein expression that contribute to the disease development. Taken together, we noted marked decreases in JAM‐2 and Collagen I levels, coupled with a significant increase in MMP‐2 expression among POP patients, indicating a shift away from normal tissue synthesis and degradation balance. This imbalance indicates a convergence of reduced tissue integrity and increased matrix breakdown, likely increasing susceptibility to POP. Our analysis further elucidates the complex interactions among these proteins, providing deeper insights into their collective impact on tissue structure and function in POP.

Importantly, our study demonstrates the significant diagnostic potential of JAM‐2, Collagen I and MMP‐2 for pelvic organ prolapse (POP). These markers could be developed into a noninvasive diagnostic panel, using vaginal swabs or biopsies, to aid in early POP detection, risk stratification and treatment planning. This combination might offer improved sensitivity and specificity compared to current diagnostic methods. However, large‐scale clinical studies are needed to validate these findings and establish standardised cut‐off values for clinical implementation.

Our study has limitations, including sample size constraints, potential confounding factors not fully accounted for and the single‐center nature of our cohort. These factors may limit the generalisability of our findings. Future research should involve multicentre studies with larger, more diverse populations. We propose longitudinal studies to assess the predictive value of JAM‐2, Collagen I and MMP‐2 in POP progression, and interventional studies to evaluate their potential as therapeutic targets. Additionally, investigating these markers in various pelvic floor disorders could provide insights into their specificity for POP.

## 5 Conclusions

In summary, our study on POP presented pioneering findings that substantially advance our understanding of the molecular basis of POP and its diagnostic landscape. By challenging the traditional emphasis on demographic factors as primary predictors of POP, our research redirected attention to the critical roles of JAM‐2, Collagen I and MMP‐2 in the pathophysiology of this condition. Our analysis not only demonstrated significant alterations in the expression of these molecules in POP patients but also established their diagnostic utility, marking a significant leap forward in the quest for reliable, noninvasive diagnostic markers. Furthermore, the correlation of molecular marker expression with POP‐Q grades offered invaluable insights into the progression of POP, providing a robust framework for future therapeutic interventions.

## Author Contributions

**Yuan Liu:** data curation (lead), validation (lead), writing – original draft (lead), writing – review and editing (lead). **Yajing Mao:** data curation (lead), validation (lead), writing – original draft (lead), writing – review and editing (lead). **Yuelin Wu:** data curation (lead), validation (lead), writing – original draft (lead), writing – review and editing (lead). **Sheng Wan:** data curation (supporting), validation (supporting), writing – original draft (supporting), writing – review and editing (supporting). **Shengyi Gu:** data curation (supporting), validation (supporting), writing – original draft (supporting), writing – review and editing (supporting). **Jing Peng:** data curation (supporting), validation (supporting), writing – original draft (supporting), writing – review and editing (supporting). **Bo Jiao:** data curation (supporting), validation (supporting), writing – original draft (supporting), writing – review and editing (supporting). **Xiaolin Hua:** data curation (lead), funding acquisition (lead), resources (lead), supervision (lead), validation (lead), writing – original draft (lead), writing – review and editing (lead).

## Ethics Statement

Informed consent was obtained from all participants prior to the surgery, and the study was approved by Shanghai First Maternity and Infant Hospital.

## Consent

The authors have nothing to report.

## Conflicts of Interest

The authors declare no conflicts of interest.

## Data Availability

The raw data supporting the conclusions of this article will be made available by the authors, without undue reservation.
